# Separation of Antioxidant Peptides from Pepsin Hydrolysate of Whey Protein Isolate by ATPS of EOPO Co-polymer (UCON)/Phosphate

**DOI:** 10.1038/s41598-017-13507-9

**Published:** 2017-10-17

**Authors:** Bin Jiang, Xiaoqing Zhang, Yongqiang Yuan, Yuxiao Qu, Zhibiao Feng

**Affiliations:** Department of Applied Chemistry, Northeast Agricultural University, Harbin, 150030 China

## Abstract

An aqueous two-phase system (ATPS) consisting of poly(ethylene glycol-ran-propylene glycol) monobutyl ether (UCON)/phosphate was developed for the separation of the antioxidant peptides from pepsin hydrolysate of Whey Protein Isolate (WPI). The efficiency of the separation was evaluated based on the DPPH radical scavenging activity, ABTS radical scavenging activity and ferric reducing antioxidant power (FRAP) of the separated peptides. The effects of some parameters on the partition of antioxidant peptides were investigated. An efficient separation of antioxidant peptides was achieved using ATPS with pH of 4.0, 4 mL of UCON solution (40%, *w*/*w*), 4 mL of KH_2_PO_4_ solution (15.5%, *w*/*w*), 2 mL of WPI hydrolysate and 0.40 g/10 mL of NaCl. Reversed-phase high-performance liquid chromatography (RP-HPLC), amino acid analyzer and matrix-assisted laser desorption ionization time-of-flight mass spectrometry (MALDI-TOF MS) were used to characterize the purified peptides separated by the ATPS. The peptides in top phase were less polar than those in bottom phase. More antioxidative and hydrophobic amino acids were extracted to the top phase of ATPS, and the peptides with the amino acid sequences with antioxidant activities moved to the top phase as well. In conclusion, antioxidant peptides were successfully separated from the WPI hydrolysate by UCON/phosphate ATPS.

## Introduction

Compared with traditional liquid-liquid extraction, an aqueous two-phase system (ATPS) is a milder extraction and separation technology that is non-toxic, non-flammable, low cost and not prone to emulsion^1^. It can also be scaled up for use on an industrial scale. Whey Protein Isolate (WPI) is one of the most abundant proteins isolated from whey, the byproduct in the manufacturing process of cheese and casein. In addition, WPI is a mixture of globular proteins that consists mainly of β-lactoglobulin (β-Lg) and α-lactalbumin (α-La). WPI is widely used as an ingredient in food because of its various functionalities and nutritional properties, such as ease of absorption and variety in its active ingredients including all the essential amino acids. Broader application of WPI is limited mainly by its high biological oxygen demand (BOD)^2^ and by the difficulty and expense of its industrial processing. Therefore, hydrolysis, a simple and effective treatment for WPI, is used to prepare it for use by the food industry.

Some bioactive peptides can be obtained from milk^3^ or hydrolysate of proteins^4^ based on the sizes and sequences of the peptides. At present, the peptides are separated by molecular exclusion chromatography and liquid chromatography (LC) and characterized by an amino acid analyzer or mass spectrometry (MS)^5^. Bah^6^ used gel permeation chromatography, OFFGEL isoelectric focusing and RP-HPLC to separate the bioactive peptides in the hydrolysate of cattle plasma using plant and fungal proteases. Then, the amino acid sequences of bioactive peptides were analyzed by LC-MS and MALDI-TOF/TOF-MS. Angiotensin-I-converting enzyme (ACE) inhibitory activity in walnut pepsin hydrolysate was measured using *in vitro* HPLC analysis, and N-terminal amino acid sequencing was used for structural identification^7^. Lassoued^8^ discovered that the highest antioxidant activity was identified in the peptide AVGAT by size-exclusion chromatography, RP-HPLC and nanoESI-LC–MS/MS. Despite the recent advances in column and instrument technology, the separations that can be achieved with LC are not sufficient to adequately resolve the sample constituents with a single-step analysis^9^. Multidimensional LC has been applied to improve the separation and reduce the sample complexity to an acceptable level prior to MS analysis^9,10^. Zenezini Chiozzi^11^ identified three novel angiotensin-converting enzyme inhibitory peptides from cauliflower by-products by multidimensional LC and bioinformatics. In addition, two novel endogenous antioxidant peptides, namely, EWFTFLKEAGQGAKDMWR and GQGAKDMWR, and two ACE-inhibitory peptides, namely, REWFTFLK and MPFLKSPIVPF, were successfully identified in donkey milk by multidimensional LC and nanoHPLC-high resolution MS^12^. However, methods based on chromatography are difficult to apply in industrial production because the techniques are operationally cumbersome, time consuming and inherently expensive. In 2014, de Souza^13^ reported for the first time the recovery of casein-derived peptides with *in vitro* inhibitory activity of ACE enzyme using ATPS formed from 1500 g mol^−1^ polyethylene glycol (PEG 1500) and sodium phosphate or potassium phosphates, meaning that ATPS can be used to specifically separate peptides with different properties. However, the enrichment efficiency of bioactive peptides from the mixed peptides in ATPS had not been studied, nor has the purification of target peptides.

The constituents of the organic phase in ATPSs generally include PEG, an ionic liquid, a hydrophilic alcohol solvent and some other polymer. Ethylene oxide and propylene oxide polymer (EOPO) is a new type of polymer that has been used to form ATPSs in recent years^14,15^. The structure of EOPO is sensitive to external conditions, such as temperature, pH, and ionic strength, and will change accordingly^16,17^. These structural changes facilitate the separation of the polymer from the material for recycling purposes^18^. ATPSs formed with EOPO have been used in the separation and purification of several substances, such as thermo-acidic amylase enzyme in red pitaya^19^; lipase in *Burkholderia cepacia* ^20^; lysozyme in hen egg white^21^; and ciprofloxacin in milk, egg, and shrimp samples^22^. In 2016, the separation of α-La and β-LG from WPI with ATPS formed by EOPO co-polymer (UCON) / phosphate was reported by Zhang. In addition, the α-La was extracted to the top phase, while the β-Lg was retained in the bottom phase^23^.

The aim of this study was to develop a rapid, simple, and inexpensive separation process for the enrichment of bioactive peptides in WPI hydrolysate. With this goal, the separation of peptides in pepsin hydrolysate of WPI by UCON / phosphate ATPS was studied for the first time, and the distribution of antioxidant peptides from the hydrolysate in ATPS was investigated.

## Materials

### Instruments

Chromatographic analysis was carried out on a Varian Prostar 210 liquid chromatograph (Agilent, USA) equipped with an ultraviolet visible detector (Agilent, USA) and a ChemStation. Solution pH was determined by an FE201EL20 pH meter (Mettler Toledo Instruments Co., Ltd., Shanghai). An AL-04 electronic analytical balance (Mettler Toledo Instruments Co., Ltd., Shanghai) was used to measure the weights of samples. Nanoporous ultrafilters with 10 kDa molecular weight cutoffs (MWCO) (Amicon® Ultra-15) were purchased from Millipore (USA). The sample was dialyzed with a 300 Da MWCO filter (Spectrum Labs, USA). A CT14D desktop high-speed centrifuge (Shanghai Techcomp Scientific Instrument Co., Ltd.) and an A150011 Type vortex mixer (Nanjing Jiajun Biological Co., Ltd.) were used for the sample treatment. The ultraviolet-visible spectrophotometer was from Beijing Purkinje General Instrument Co., Ltd. The sample was characterized by a Hitachi L-8800 amino acid analyzer (Hitachi, Japan) and a Microflex MALDI-TOF mass spectrometer (Bruker Daltonics, USA).

### Reagents

WPI containing 91.5% protein was obtained from Hilmar (USA). Pepsin 1:10000 and poly(ethylene glycol-ran-propylene glycol) monobutyl ether (UCON, Mn ≈ 3900) were purchased from Sigma (USA). 2,2′- azinobis(3-ethylbenzothiazoline-6-sulphonic acid) (ABTS) (Shanghai Aladdin Bio-Chem Technology Co. Ltd.), 2,2-diphenyl-1-picrylhydrazyl (DPPH) (Sigma, USA) and Folin–Ciocalteu phenol reagent (Beijing Solarbio Science & Technology Co., Ltd.) were used for the sample analysis. Acetonitrile and trifluoroacetic acid (TFA) were chromatographically pure (Dikma, USA). K_2_HPO_4_, KH_2_PO_4_, NaCl and other reagents were analytical reagent grade.

## Methods

### Preparation of WPI hydrolysate

WPI was dissolved in water at a concentration of 5% (*w/w*), and the WPI solution was hydrolyzed at pH 2.0. The pH of the solution was adjusted using 1.0 mol/L HCl. Pepsin was added to the WPI solution, which was then maintained at 37 °C for 4 h. After hydrolysis, the samples were placed in a water bath at 100 °C for 10 min to deactivate the enzyme^24,25^. After being cooled to room temperature, the deactivated samples were centrifuged (8400 x *g*, 30 min) to remove the unhydrolyzed WPI solids, and then the supernatant was passed through ultrafilters with nanoporous filters (10 kDa MWCO) at 4500 x *g* for 30 min in order to remove any WPI solids that were not hydrolyzed but were soluble in water^26^. The ultrafiltrate was stored at −20 °C until further analysis.

### Preparation of ATPSs

ATPSs were prepared in 10 mL graduated centrifuge tubes by mixing the appropriate amount of 40% (*w/w*) stock solution of UCON, 15.5% (*w/w*) stock solution of phosphate and solid NaCl to obtain a final volume of 8 mL. WPI hydrolysate (2 mL) was then added to each system. The compounds were mixed using a vortex mixer and centrifuged at 700 x *g* for 20 min to induce phase separation. The mixtures were stored at 4 °C for 12–16 h^23^. The volumes of the top and bottom phase were measured, and then both phases were put aside for analysis.

### Determination of the distribution of peptides in ATPSs

The Folin–Ciocalteu phenol reagent^27^ was used to determine the concentrations of peptides in the hydrolysate and both phases of the ATPSs. The separation parameters were calculated using the following formulae (1, 2 and 3):

1 $$R={V}_{{\rm{T}}}/{V}_{{\rm{B}}}$$

2 $${K}_{{\rm{p}}}={C}_{{\rm{T}}}/{C}_{{\rm{B}}}$$

3 $$Y=(CV/{C}_{0}{V}_{0})\times 100 \% $$

where *R* is the ratio of the volumes of the phases, *K*_p_ is the distribution coefficient, *Y* is the extraction efficiency, *C* is the concentration of peptides in the top or bottom phase of the ATPS, *V* is the top or bottom phase volume, *C*_0_ is the concentration of the peptides in the WPI hydrolysate that was added to the ATPS, and *V*_0_ is the volume of the WPI hydrolysate added to the ATPS.

### Effects of the composition of ATPSs on the separation of antioxidant peptides

The DPPH radical scavenging activity, ABTS radical scavenging activity and ferric reducing antioxidant power (FRAP) of the peptides were investigated to explore the effect of different ATPS compositions on the distribution of antioxidant peptides. The experimental method was taken from Venkatesan^28^.

### Purification of peptides

The top phase and bottom phase were mixed with ethanol in 1:10 ratios (*V*_phase_:*V*_ethanol_) to remove the polymer-UCON, respectively. After stirring at 4 °C for 4 h, the solution was centrifuged at 8400 x *g* for 30 min. Then, the precipitated mixture of peptides and salts was collected. After stirring and centrifuging the sample three times, the combined solids were dissolved in 3–5 mL of ultrapure water and dialyzed (300 Da) for 48 h. A lyophilizer was used to get the solid particles called the purified peptides from the top phase and purified peptides from the bottom phase. The WPI hydrolysate was directly dialyzed (300 Da) and lyophilized to isolate the purified peptides from WPI hydrolysate.

### RP-HPLC analysis of the peptides

RP-HPLC was used to analyze the peptides in the WPI hydrolysate and in both phases before and after purification. The conditions for RP-HPLC were as follows: Agilent C18 column (150 mm × 4.6 mm, 5 μm), 0.1% aqueous TFA as mobile phase A, 0.1% TFA in acetonitrile as mobile phase B, flow rate of 1.00 mL/min and detection wavelength of 214 nm. Gradient elution was programmed as follows: the concentration of mobile phase B was increased from 2% to 40% over 60 min.

### Amino acids analysis for purified peptides

An automatic amino acid analyzer was used for quantitative determination of amino acids according to the GB/T 5009.124-2003^29^.

### MALDI-TOF MS analysis for purified peptides

The solid purified peptides were dissolved in ultrapure water (0.2 mg/mL). A saturated α-cyano-4-hydroxy cinnamic acid (CHCA) solution was prepared by dissolving 100 mg of CHCA in 10 mL of 1:1 acetonitrile/ultra-pure water (containing 0.1% TFA). The sample was then mixed with the saturated CHCA solution in a 1:1 ratio for analysis. A Bruker Microflex linear MALDI-TOF mass spectrometer was used for these experiments. All mass spectra were obtained using 337 nm radiation over an *m*/*z* range of 500–4000.

### Data availability

The datasets generated and analyzed during the current study are available from the corresponding author upon reasonable request.

## Results and Discussion

### Effect of pH on separation of antioxidant peptides

**Figure 1:**
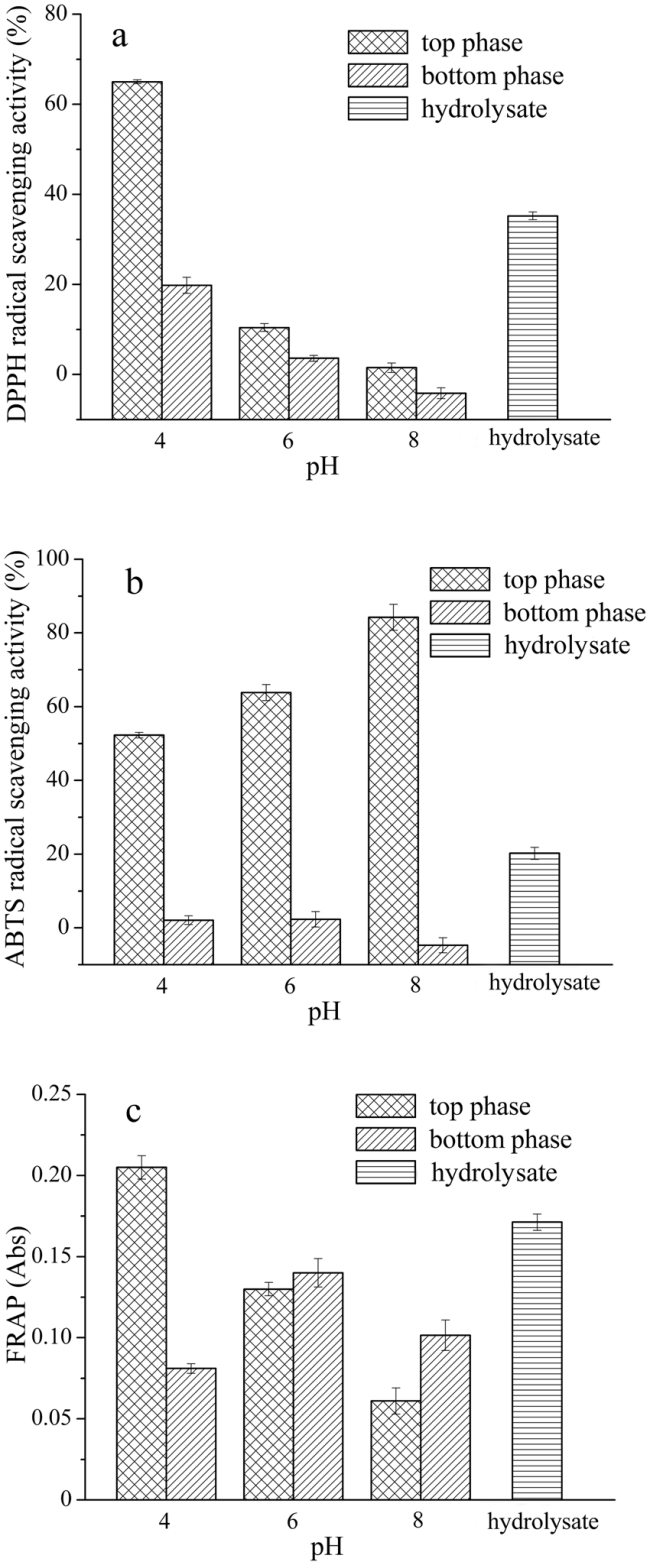
Effect of pH on the separation of antioxidant peptides. (**a**) DPPH radical scavenging activity; (**b**) ABTS radical scavenging activity; (**c**) ferric reducing antioxidant power (FRAP).

ATPSs containing 4 mL of 40% (*w/w*) UCON solution, 2 mL of 49.35 mg/mL WPI hydrolysate, 0.40 g/10 mL of NaCl solid, and 4 mL of a 15.5% (*w/w*) phosphate solution at a specific pH (pH = 4, 6 and 8) were prepared to study the effect of pH on the separation of antioxidant peptides. The results are shown in Table 1 and Fig. 1.

**Table 1:** Effect of pH on the separation of peptides.

Aqueous two-phase system variable	Top phase		Bottom phase		Volume ratio *R*	Partition coefficient *K*_p_
	Volume *V*_T_ mL	Yield *Y*_T_ (%)	Volume *V*_B_ mL	Yield *Y*_B_ (%)		
UCON/KH_2_PO_4_ (pH = 4.00)	4.0 ± 0.10^c^	38.95 ± 0.56^c^	6.0 ± 0.10^a^	54.04 ± 0.38^a^	0.67 ± 0.03^c^	1.08 ± 0.04^b^
UCON/K_2_HPO_4_-KH_2_PO_4_ (pH = 6.00)	4.6 ± 0.00^b^	49.47 ± 0.83^b^	5.4 ± 0.00^b^	47.65 ± 0.74^b^	0.85 ± 0.00^b^	1.14 ± 0.01^a^
UCON/K_2_HPO_4_ (pH = 8.00)	5.3 ± 0.10^a^	54.40 ± 0.68^a^	4.7 ± 0.10^c^	40.67 ± 0.58^c^	1.13 ± 0.05^a^	1.19 ± 0.03^a^

^1^The data (mean ± standard deviation) derived from 3 replicates as described in the Materials and Methods section.

^2^The difference between the mean values with the same superscript letter in the same column was significant at a 95% confidence interval.

According to the results presented in Table 1, *R*, *Y*_T_ and *K*_p_ increased with increasing pH, but *Y*_B_ decreased. Figure 1(a) shows that the DPPH radical scavenging ability of peptides in both phases increased to the same extent as the pH decreased, and the difference in the DPPH radical scavenging rates of the peptides in the top and bottom phase increased. At pH = 6 or 8, the DPPH radical scavenging activities of the peptides in both phases were lower than the activity in the WPI hydrolysate. This is likely due to some peptides being denatured at a high pH. At pH 4, the DPPH radical scavenging activity of the peptides in the top phase was far higher than that of the peptides in the bottom phase and the WPI hydrolysate, and at the same time, the extraction yield of the peptides in the top phase was lower than that of the proteins in the bottom phase. In addition, as shown in Fig. 1(b), the ABTS radical scavenging activity of the peptides in the top phase was significantly higher than that of the peptides in the bottom phase and the WPI hydrolysate at all three pH levels, although the ABTS radical scavenging activity of peptides in the top phase increased with increasing pH. The results indicate that the ATPS was sufficiently selective for antioxidant peptides, and the selectivity was not affected by pH. Therefore, the antioxidant peptides tended to remain in the top phase even at pH 4.

At pH 6 and 8, the ferric reducing antioxidant power (FRAP) of the peptides in the top phase was weaker than that of the peptides in the bottom phase (Fig. 1(c)), implying that the desired separation of antioxidant peptides was not achieved. In contrast, when the pH was 4, the FRAP of the peptides in the top phase was greater than that of the peptides in the bottom phase, which meant that the antioxidant peptides were distributed in the top phase. The FRAP was closely related to the amount of H· supplied by antioxidants, that is to say, H· could quench free radicals^30^. Therefore, when the pH of the ATPS was 4, the top phase contained more antioxidant peptides with the ability to generate H·, which can reduce Fe^3+^ to Fe^2+^. In summary, acidic conditions (pH = 4) favored the separation of antioxidant peptides in WPI hydrolysate.

### Effect of the ratio of the volumes UCON and KH_2_PO_4_ on the separation of antioxidant peptides

**Figure 2:**
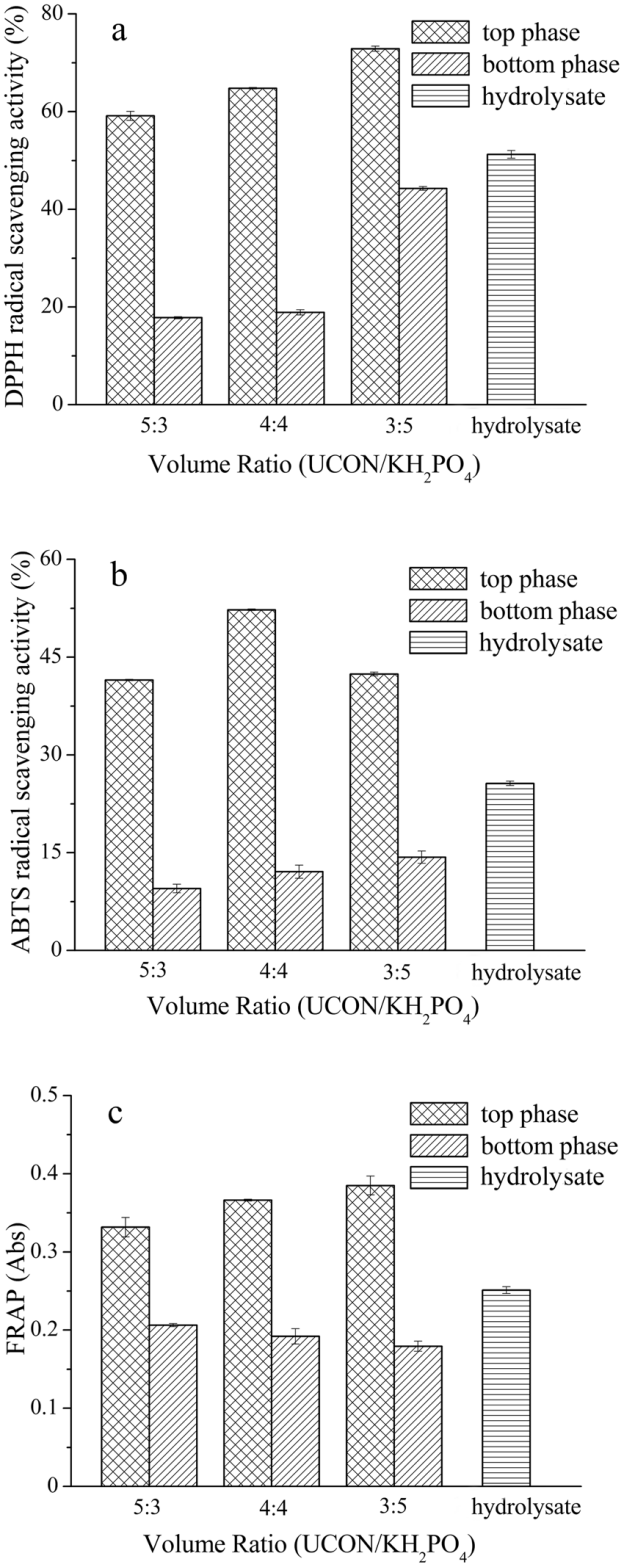
Effect of the ratio of the volumes of UCON and KH_2_PO_4_ on the separation of antioxidant peptides. (**a**) DPPH radical scavenging activity; (**b**) ABTS radical scavenging activity; (**c**) ferric reducing antioxidant power (FRAP).

The volume ratio is an important factor in the separation of antioxidant peptides in ATPSs. ATPSs containing different ratios of volumes of UCON/KH_2_PO_4_ (5:3, 4:4 and 3:5) were used to investigate the effects of the composition of the system on the distribution efficiency of antioxidant peptides. WPI hydrolysate (2 mL, 49.35 mg/mL) was added to the system, and the NaCl concentration was 0.40 g/10 mL (Table 2). The antioxidant activities of the top and bottom phases were determined. The results are shown in Fig. 2.

**Table 2:** Effect of the ratio of the volumes of UCON and KH_2_PO_4_ on the separation of peptides.

Aqueous two-phase system variable	Top phase		Bottom phase		Volume ratio *R*	Partition coefficient *K*_p_
	Volume *V*_T_ mL	Yield *Y*_T_ (%)	Volume *V*_B_ mL	Yield *Y*_B_ (%)		
5 mL:3 mL	5.8 ± 0.00^a^	49.54 ± 0.56^a^	4.2 ± 0.00^b^	36.34 ± 0.30^c^	1.38 ± 0.00^a^	0.99 ± 0.13^a^
4 mL:4 mL	4.0 ± 0.10^b^	38.95 ± 0.56^b^	6.0 ± 0.10^a^	54.04 ± 0.38^b^	0.67 ± 0.03^b^	1.08 ± 0.04^a^
3 mL:5 mL	3.8 ± 0.20^b^	38.73 ± 0.72^b^	6.2 ± 0.20^a^	62.00 ± 0.80^a^	0.61 ± 0.05^b^	1.02 ± 0.02^a^

^1^The data (mean ± standard deviation) derived from 3 replicates as described in the Materials and Methods section.

^2^The difference between the mean values with the same superscript letter in the same column was significant at a 95% confidence interval.

*R* and *Y*_T_ decreased, *Y*_B_ increased, and *K*_p_ was almost constant as the amount of UCON decreased (Table 2). When UCON and KH_2_PO_4_ were present in a 4:4 *V*/*V* ratio, the difference between the DPPH, ABTS radical scavenging activity and FRAP of the peptides in the two phases was larger than it was when other ratios were used (Fig. 2). Considering these factors, a 4:4 *V*/*V* ratio of UCON/K_2_HPO_4_ of was selected as the optimal conditions for the separation of the antioxidant peptides from WPI hydrolysate.

### Effect of NaCl content on the separation of antioxidant peptides

In the ATPSs, NaCl has a stronger salting-out ability than other salts such as KCl, LiCl, CaCl_2_ and NaBr, and it can therefore fully distribute the biomolecules in ATPS to achieve complete separation^31^. The salting-out ability increased with increasing amounts of NaCl. However, possibly because of the high ionic strength, the proteins denatured and precipitated at high NaCl concentrations. NaCl was added to an ATPS of UCON/KH_2_PO_4_ (4:4, *V*/*V*) to give a final concentration of 0.40 g/10 mL, and the extraction efficiencies of antioxidant peptides in the system were investigated. A separate ATPS of UCON/KH_2_PO_4_ (4:4, *V*/*V*) without NaCl was used for comparison.

As shown in Table 3, the volume ratio and *Y*_T_ decreased and *Y*_*B*_ increased when NaCl was added to the system. As indicated by the data in Table 4, the antioxidant activity of the peptides in the top phase significantly increased when NaCl was added, and the activities were much greater than those of the peptides in the bottom phase. The results showed that more antioxidant peptides were extracted into the top phase, and the enrichment of the antioxidant peptides in the top phase of the ATPS was enhanced in the presence of NaCl. Therefore, the addition of NaCl was used to improve the separation of the antioxidant peptides in the WPI hydrolysate. Based on three single-factor experiments and the antioxidant activities of the top and bottom phases, the optimized ATPS at a pH of 4, with a 4:4 *V*/*V* ratio of 40% (*w/w*) UCON solution to 15.5% (*w/w*) KH_2_PO_4_ solution, and the addition of 0.40 g/10 mL NaCl was selected for the separation of the antioxidant peptides from the WPI hydrolysate.

**Table 3:** Effect of NaCl on the separation of peptides.

Aqueous two-phase system variable	Top phase		Bottom phase		Volume ratio *R*	Partition coefficient *K*_p_
	Volume *V*_T_ mL	Yield *Y*_T_ (%)	Volume *V*_B_ mL	Yield *Y*_B_ (%)		
NaCl = 0 g/10mL	6.5 ± 0.00^a^	52.36 ± 0.87^a^	3.5 ± 0.00^b^	38.59 ± 0.60^b^	1.86 ± 0.00^a^	0.74 ± 0.06^b^
NaCl = 0.40 g/10mL	4.0 ± 0.10^b^	38.95 ± 0.56^b^	6.0 ± 0.10^a^	54.04 ± 0.38^a^	0.67 ± 0.03^b^	1.08 ± 0.04^a^

^1^The data (mean ± standard deviation) derived from 3 replicates as described in the Materials and Methods section.

^2^The difference between the mean values with the same superscript letter in the same column was significant at a 95% confidence interval.

**Table 4:** Effect of NaCl on the separation of antioxidant peptides.

Aqueous two-phase system variable		DPPH (%)	ABTS	FRAP
NaCl = 0 g/10mL	Top phase	32.93 ± 0.07^c^	42.51 ± 0.91^b^	0.12 ± 0.00^e^
	Bottom phase	22.04 ± 0.28^d^	25.14 ± 0.16^c^	0.29 ± 0.01^b^
NaCl = 0.40 g/10mL	Top phase	64.77 ± 0.13^a^	52.04 ± 0.11^a^	0.37 ± 0.02^a^
	Bottom phase	18.91 ± 0.53^e^	14.47 ± 0.26^e^	0.19 ± 0.01^d^
	WPI hydrolysate	49.53 ± 0.52^b^	23.45 ± 0.19^d^	0.24 ± 0.00^c^

^1^The data (mean ± standard deviation) derived from 3 replicates as described in the Materials and Methods section.

^2^The difference between the mean values with the same superscript letter in the same column was significant at a 95% confidence interval.

### RP-HPLC analysis of peptides

**Figure 3:**
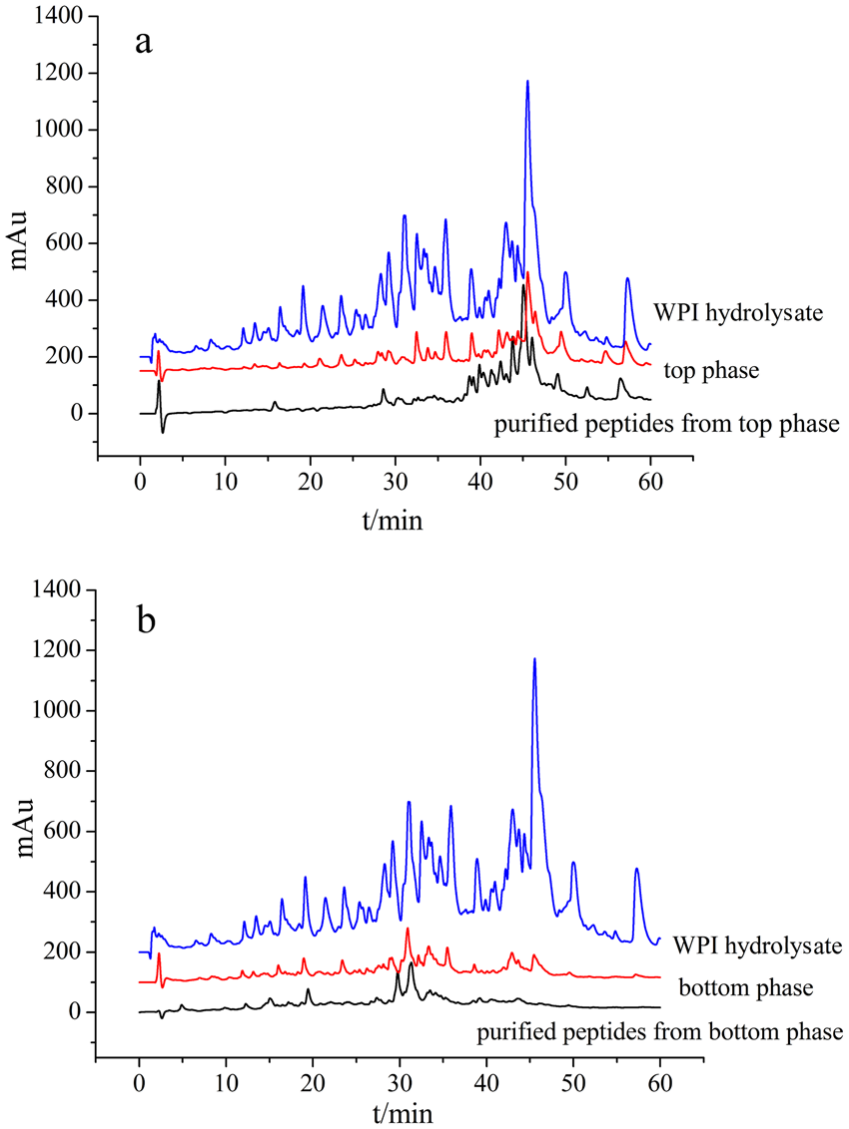
RP-HPLC analysis of the peptides. (**a**) RP-HPLC chromatograms of the WPI hydrolysate, top phase and purified peptides from the top phase, b: RP-HPLC chromatograms of the WPI hydrolysate, bottom phase and purified peptides from the bottom phase.

Under the optimized conditions described above, the peptides in the WPI hydrolysate and in both phases before and after purification were measured by RP-HPLC. The retention times and peak areas of the samples were recorded. The obtained chromatograms are shown in Fig. 3.

In the separation of the antioxidant peptides by ATPS, unlike in the chromatogram of the WPI hydrolysate, the retention times of the peaks in the chromatogram of the top phase ranged from 40 min to 60 min, and the retention times of the peaks in the chromatogram of the bottom phase were all approximately 30 min; these retention times responded to the position of the peptides from the hydrolysate (Fig. 3). At shorter retention times, the efflux from the C18 column in RP-HPLC analysis comprised the hydrophilic and higher polar peptides that were distributed in the bottom phase. At longer retention times, the efflux comprised the hydrophobic and lower polar peptides that were extracted into the top phase.

Xie reported that the highly hydrophobic peptides from casein had excellent bioavailability and remaining antioxidant activity^32^. Bamdad reported that barley hordein fractions separated based on surface hydrophobicity with strong antioxidant activity^33^. Rousselot-Pailley^34^ proved that the polarity of the protein environment was crucial for the selectivity of sulfide oxidation by an artificial metalloenzyme studied. Silk fibroin is rich in non-polar amino acids, which account for approximately 69.08% of the total amino acids; however, the product of *in vitro* digestion of silk fibroin exhibited high DPPH free radical scavenging activity, Fe^2+^ chelating ability and reducing power^35^. Moreover, the antioxidant activity of the peptides in the top phase was substantially higher than that of the peptides in the bottom phase. Therefore, the retention times of peptides with antioxidant activities were between 40 min and 60 min. There were two small peaks in the chromatograph of the bottom phase between 40 min and 50 min, which might be related to the small antioxidant activity observed in the bottom phase. Although ATPS is a mild extraction and separation technique, there are still some losses during extraction. A thin middle layer was produced due to salting out effects that trapped some impure proteins between the two phases^36^. These impure proteins were likely to be highly polar or hydrophilic peptides. Therefore, some peaks that should have appeared before 40 min in the chromatogram of the top phase were not observed. This was also explained and verified by the sum of the top and bottom phase extraction efficiency (*Y*) being less than 100% (approximately 90%) (Tables 1, 2 and 3). Since the antioxidant peptides were weakly polar or non-polar and hydrophobic, and the retention times of the antioxidant peptides were between 40 min and 60 min, it was assumed that the lost peptides did not have antioxidant activity. These results proved that the ATPS of UCON/KH_2_PO_4_ could separate antioxidant peptides from the WPI hydrolysate based on the polarity or hydrophobicity of the peptides.

In comparing the retention times of the purified peptides to those of the peptides in the phases, the retention times of the purified peptides from top phase ranged from 40 min-60 min, and those times matched the retention times of the corresponding peptides still in the top phase (Fig. 3(a)). The retention times in the chromatograms of the purified peptides from the bottom phase were approximately 30 min, which matched the retention times of the corresponding peptides still in the bottom phase (Fig. 3(b)). A few of the peaks in the chromatograms of the top and bottom phases between 30 min and 40 min were not observed in the chromatograms of the purified peptides due to losing a small amount of peptide during purification with ethanol. Because of the temperature sensitivity and the large molecular weight (Mn ≈ 3900) of the UCON polymer, the purification was difficult. Moreover, UCON was soluble in ethanol, and thus, even if some peptides were susceptible to structural changes in ethanol media^37^, the peptides had to ultimately be purified from the ATPS using ethanol at 4 °C. A better method for the purification of antioxidant peptides away from ATPS is a goal of further studies.

### Amino acid analysis of the purified peptides

The amino acids in the WPI hydrolysate and the purified peptides from the top and bottom phases were examined with an amino acid analyzer, and the comparison of the types and content of the amino acids are shown in Table 5.

**Table 5:** Comparison of the types and content of amino acids.

Types of amino acids	The contents of amino acids in the WPI hydrolysate (%)	The contents of amino acids in the purified peptides from top phase (%)	The change of contents of amino acids (%)	The contents of amino acids in the purified peptides from bottom phase (%)	The change of contents of amino acids (%)
Aspartic acid	11.16	10.85	−0.31	12.35	+1.19
Threonine	8.78	4.55	−4.23	9.07	+0.29
Serine	5.27	3.62	−1.65	5.83	+0.56
Glutamic acid	16.55	17.55	+1	21.22	+4.67
Glycine	2.37	1.18	−1.19	1.60	−0.77
Alanine	4.63	4.39	−0.24	3.13	−1.5
Cystine	1.73	5.61	+3.88	1.71	−0.02
Valine	5.45	5.69	+0.24	4.13	−1.32
Methionine	2.12	1.18	−0.94	1.50	−0.62
Isoleucine	6.21	5.69	−0.53	8.34	+2.12
Leucine	10.93	9.31	−1.62	6.27	−4.66
Tyrosine	3.25	5.24	+1.99	1.97	−1.28
Phenylalanine	2.56	6.95	+4.39	2.12	−0.44
Lysine	9.07	11.34	+2.27	8.84	−0.23
Histidine	1.52	2.44	+0.92	1.32	−0.2
Argnine	1.96	0.96	−1	1.41	−0.55
Proline	6.43	3.45	−2.98	9.19	+2.76

Some amino acids with antioxidant activities, such as Cystine, Histidine, Tyrosine, Lysine and Arginine, as well as hydrophobic amino acids, such as Valine, Leucine, Isoleucine and Phenylalanine, were indispensable active ingredients in the antioxidant peptides, and the antioxidant activities of the peptides were determined based on the types and sequences of amino acids^38–40^. The types of amino acids in the purified peptides from the top/bottom phase and the WPI hydrolysate were the same, but they were present in different amounts (Table 5). In addition, the content of Cystine, Histidine, Tyrosine, Lysine, Valine and Phenylalanine, which have antioxidant activities or are hydrophobic, was significantly higher in the purified peptides from the top phase than they were in the WPI hydrolysate. The content of these amino acids was significantly lower in the purified peptides from the bottom phase than they were in the WPI hydrolysate. The results showed that the antioxidative and hydrophobic amino acids were extracted into the top phase from WPI hydrolysate by UCON/KH_2_PO_4_ ATPS, which enhanced the antioxidant activity of peptides in the top phase of the ATPS.

### MALDI-TOF MS analysis for purified peptides

**Figure 4:**
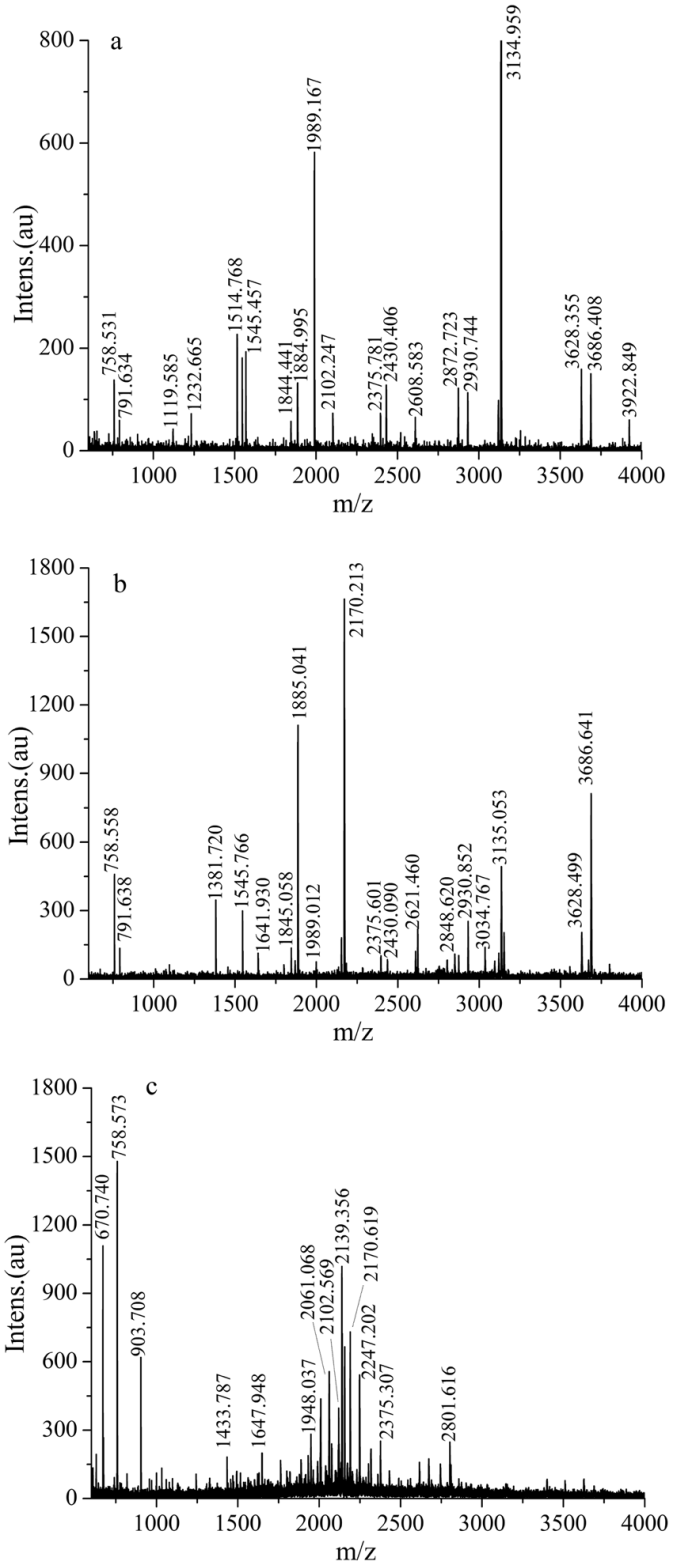
MALDI-TOF MS analysis of purified peptides. (**a**) MALDI-TOF spectrum of purified peptides from the WPI hydrolysate; (**b**) MALDI-TOF spectrum of purified peptides from the top phase; (**c**) MALDI-TOF spectrum of purified peptides from the bottom phase.

The purified peptides from the WPI hydrolysate and the top and bottom phases were analyzed using MALDI-TOF MS, and the results are shown in Fig. 4. Among the components of the WPI, α-La and β-Lg account for approximately 80% of the total protein content. The complete amino acid sequences with identified peptide chains of α-La and β-Lg in bovine milk were selected, and the potential cleavage sites for the hydrolysis of WPI into peptides by pepsin were analyzed by UniProt (http://www.uniprot.org/) and PeptideCutter^41^. The UniProtKB of α-La is P00711, and the UniProtKB of β-Lg is P02754. The molecular weights of the peptides were calculated and analyzed by PeptideMass^42^ and BIOPEP (http://www.uwm.edu.pl/biochemia/index.php/pl/biopep/)^43^ and compared with the MALDI-TOF MS data to identify the amino acid sequences of the peptides in the top and bottom phases of the ATPS. The amino acid sequences of peptides from different phases are shown in Table 6.

**Table 6:** Amino acid sequences of peptides from different phases.

The source of the peptides	Protein fragment	observed m/z	calculated m/z	Amino acid sequences	Peptide having antioxidant activity
bottom phase	β-Lg 27–32	670.74	672.7795	DIQKVA	
hydrolysate	β-Lg 153–159	758.531	757.9285	DKALKAL	
top phase	β-Lg 173–178	758.558	757.8163	EEQCHI	
hydrolysate	β-Lg 56–61	791.634	793.875	RVYVEE	
top phase	β-Lg 144–150	791.638	789.7943	VDDEALE	
top phase	β-Lg 27–38	1381.72	1380.5633	DIQKVAGTWYSL	WY
					WYS
hydrolysate	β-Lg 22–35	1545.457	1547.8328	TMKGLDIQKVAGTW	
top phase	β-Lg 82–96	1641.930	1642.0331	CAQKKIIAEKTKIPA	
hydrolysate	β-Lg 104–118	1844.441	1842.079	NENKVLVLDTDYKKY	VLVLDTDYK
top phase		1845.058			
hydrolysate	β-Lg 82–98	1884.995	1888.3423	CAQKKIIAEKTKIPAVF	KTKIPAVF
top phase		1885.041			IPAVF
hydrolysate	β-Lg 18–35	1989.167	1989.3606	IVTQTMKGLDIQKVAGTW	
top phase		1989.012			
bottom phase	β-Lg 42–50	903.708	903.9847	ASDISLLDA	KTKIPAVF
	β-Lg 91–98		903.1327	KTKIPAVF	IPAVF
bottom phase	β-Lg 78–90	1433.787	1432.6116	ENGECAQKKIIAE	
bottom phase	α-La 45–58	1647.948	1646.7708	WVCTTFHTSGYDTQ	
	β-Lg 134–148		1648.8233	ACQCLVRTPEVDDEA	
hydrolysate	α-La32–50	2102.247	2101.4244	KDLKGYGGVSLPEWVCTTF	
bottom phase	β-Lg17–35	2102.569	2102.5200	LIVTQTMKGLDIQKVAGTW	
bottom phase	α-La 45–62	2061.068	2058.2723	WVCTTFHTSGYDTQAIVQ	
bottom phase	β-Lg 84–102	2139.356	2141.6256	QKKIIAEKTKIPAVFKIDA	KTKIPAVF
	β-Lg 91–109		2141.5822	KTKIPAVFKIDALNENKVL	IPAVF
bottom phase	α-La51–69	2247.202	2144.1719	HTSGYDTQAIVQNNDSTEY	
hydrolysate	β-Lg 49–58	1119.585	1119.2426	DAQSAPLRVY	YVEEL^43^
	β-Lg 43–53		1119.1936	SDISLLDAQSA	
hydrolysate	β-Lg 48–58	1232.665	1232.402	LDAQSAPLRVY	
	α-La 70–79		1233.455	GLFQINNKIW	
hydrolysate	α-La116–128	1514.768	1515.7545	DKVGINYWLAHKA	

In addition to α-La and β-Lg, there were other proteins in the WPI. Some amino acid sequences in the WPI hydrolysate were not identified and could be attributed to those protein impurities. For this reason, not all the amino acid sequences were elucidated by MALDI-TOF MS. Furthermore, peptides from the hydrolysis of β-LG were prone to structural changes in ethanol. The different numbers of peptides generated by pepsin in the WPI could be a result of the structural changes in the peptides during purification with ethanol. These structural changes complicated the analysis of the WPI hydrolysate. These factors also caused some of the MALDI-TOF MS peaks of the top and bottom phases to not correspond to those of the hydrolysate (Fig. 4).

Dávalos^44^ studied the antioxidant peptides obtained from the pepsin hydrolysate of egg white. The peptides with molecular weights of approximately 3 KDa were the main contributors to the antioxidant activity. Hernandez-Ledesma^45^ used HPLC-MS/MS to identify the antioxidant peptides obtained from the hydrolysate of α-La and β-Lg, and the results also showed that the molecular weight of the antioxidant peptides was approximately 3 KDa. Almost all the peptides with molecular weights of approximately 3 KDa were found in the top phase, which caused the antioxidant activity of the peptides in the top phase to be higher than that of the peptides in the bottom phase (Fig. 4). Ha^46^ studied the antioxidant peptides obtained from the hydrolysate of skim milk powders, and some of the peptides of the peptide having antioxidant activity potentially possessed antioxidant activity. In this experiment, the special DIQKVAGTWYSL, NENKVLVLDTDYKKY and CAQKKIIAEKTKIPAVF sequences found in the peptides of the top phase contained the WY, WYS, WYSL^43^, VAGTWY, VAGTWY, VAGTWYLS^47^, VLVLDTDYK^48^, KTKIPAVF^46^ and IPAVF^48^ peptides, which have antioxidant activity (Table 6). This may be another reason that the antioxidant activities of the peptides in the top phase were higher than those of the peptides in the bottom phase.

## Conclusions

In this study, antioxidant peptides from pepsin hydrolysate of WPI were separated by an ATPS containing UCON/phosphate. Under optimum conditions, including pH = 4, 4:4 volume ratio of 40% (*w*/*w*) UCON solution and 15.5% (*w/w*) KH_2_PO_4_ solution, 2 mL of 49.35 mg/mL WPI hydrolysate and 0.40 g/10 mL NaCl, the extraction efficiency (*Y*_T_) of the peptides was 38.95%, while the antioxidant activities of the peptides in the top phase were much higher than those of the peptides in the bottom phase and in the hydrolysate. According to RP-HPLC analysis of the peptides, the ATPS of UCON/KH_2_PO_4_ separated antioxidant peptides from the WPI hydrolysate based on the polarity or hydrophobicity of the peptides. Based on MALDI-TOF MS analysis of the purified peptides, the top phase of the ATPS was mainly composed of peptides with molecular weights of approximately 3 KDa with antioxidant activity and peptides that contained amino acid sequences with antioxidant activity. In summary, the separation of antioxidant peptides from pepsin hydrolysate of WPI was achieved by ATPS of UCON/phosphate. Despite some challenges in this purification method, the goal of isolating a portion of peptides from ATPS was achieved to a degree.
